# Formation of the chaperonin complex studied by 2D NMR spectroscopy

**DOI:** 10.1371/journal.pone.0187022

**Published:** 2017-10-23

**Authors:** Toshio Takenaka, Takashi Nakamura, Saeko Yanaka, Maho Yagi-Utsumi, Mahesh S. Chandak, Kazunobu Takahashi, Subhankar Paul, Koki Makabe, Munehito Arai, Koichi Kato, Kunihiro Kuwajima

**Affiliations:** 1 Okazaki Institute for Integrative Bioscience and Institute for Molecular Science, National Institutes of Natural Sciences, Myodaiji, Okazaki, Aichi, Japan; 2 Department of Functional Molecular Science, School of Physical Sciences, the Graduate University for Advanced Studies (Sokendai), Myodaiji, Okazaki, Aichi, Japan; 3 Department of Physics, Graduate School of Science, the University of Tokyo, Bunkyo-ku, Tokyo, Japan; 4 Graduate School of Science and Engineering, Yamagata University, Yonezawa, Yamagata, Japan; 5 Department of Life Sciences, Graduate School of Arts and Sciences, the University of Tokyo, Meguro-ku, Tokyo, Japan; 6 School of Computational Sciences, Korea Institute for Advanced Study (KIAS), Dongdaemun-gu, Seoul, Korea; George Washington University, UNITED STATES

## Abstract

We studied the interaction between GroES and a single-ring mutant (SR1) of GroEL by the NMR titration of ^15^N-labeled GroES with SR1 at three different temperatures (20, 25 and 30°C) in the presence of 3 mM ADP in 100 mM KCl and 10 mM MgCl_2_ at pH 7.5. We used SR1 instead of wild-type double-ring GroEL to precisely control the stoichiometry of the GroES binding to be 1:1 ([SR1]:[GroES]). Native heptameric GroES was very flexible, showing well resolved cross peaks of the residues in a mobile loop segment (residue 17–34) and at the top of a roof hairpin (Asn51) in the heteronuclear single quantum coherence spectra. The binding of SR1 to GroES caused the cross peaks to disappear simultaneously, and hence it occurred in a single-step cooperative manner with significant immobilization of the whole GroES structure. The binding was thus entropic with a positive entropy change (219 J/mol/K) and a positive enthalpy change (35 kJ/mol), and the binding constant was estimated at 1.9×10^5^ M^−1^ at 25°C. The NMR titration in 3 mM ATP also indicated that the binding constant between GroES and SR1 increased more than tenfold as compared with the binding constant in 3 mM ADP. These results will be discussed in relation to the structure and mechanisms of the chaperonin GroEL/GroES complex.

## Introduction

The *Escherichia coli* (*E*. *coli*) chaperonin GroEL and its partner co-chaperone GroES provide the best-characterized molecular chaperone system, and the binding of GroES to and its release from GroEL, coupled with ATP binding and hydrolysis, are essential steps in the chaperonin cycle, by which efficient folding of a stringent substrate protein takes place inside the chaperonin cavity formed by the GroEL/GroES complex [1–3]. Therefore, studies on the thermodynamic binding parameters, including the binding constant (*K*_b_), the enthalpy change (Δ*H*) and the entropy change (Δ*S*), of the GroES–GroEL binding are important for fully elucidating the molecular mechanisms of the chaperonin function. However, rather surprisingly, thermodynamic studies to evaluate the above binding parameters have not yet been reported for the GroEL/GroES chaperonin system, although a large number of biophysical studies on the chaperonin molecular mechanisms have been reported, as nicely summarized in recent review articles [1–4]. As far as we are aware, only the *K*_b_ values obtained by direct measurements [5, 6] or by indirect measurements [7, 8] have so far been reported.

Here, we thus studied the interaction between GroES and a single ring mutant (SR1) of GroEL by the NMR titration of ^15^N-labeled GroES with SR1 at three different temperatures, 20, 25 and 30°C, in the presence of 3 mM ADP at pH 7.5. We used SR1 instead of wild-type double-ring GroEL, because wild-type GroEL forms two different types of complexes with GroES, a 1:1 ([GroEL]:[GroES]) "bullet"-shaped complex and a 1:2 "football"-shaped complex, depending on the solution conditions [1, 9, 10], and hence the stoichiometry of the GroES binding to GroEL may be varied. By using SR1, we can precisely control the stoichiometry of the GroES binding to be 1:1 ([SR1]:[GroES]). SR1 contains four mutations (R452E, E461A, S463A and V464A), which are located at the major contact sites between the two rings of GroEL. It therefore forms the single-ring structure under physiological conditions, and stably binds to one GroES heptamer in the presence of ADP or ATP [11]. Because of the absence of the opposite ring, the substrate protein encapsulated by the SR1/GroES complex is very hard to release. Nevertheless, substrates (rhodanese and green fluorescent protein) can complete the folding to the native state in the presence of ATP while remaining encapsulated within the cavity of the SR1/GroES complex, indicating that the SR1/GroES complex has a partial chaperone activity to promote the folding of the substrate proteins [12]. Interestingly, by introducing a mutation which reduces the affinity between SR1 and GroES, the SR1/GroES complex becomes fully functional [13–15]. Therefore, the thermodynamic binding parameters between GroES and SR1 are highly relevant for understanding the chaperonin mechanisms, and provide valuable information about the GroES binding to GroEL.

In the present study, the 2D ^1^H–^15^N heteronuclear single quantum coherence (HSQC) spectra of ^15^N-labeled GroES in the native heptameric state exhibited well-resolved cross peaks of the residues in a mobile loop segment (residues 17–34) and at the top of a roof hairpin (Asn51), indicating that not only the mobile loop but also the opposite site (Asn51) was highly flexible in the native GroES. The binding of SR1 to GroES caused these cross peaks to disappear simultaneously, and thus we assumed that the binding occurred in a single-step cooperative manner with significant immobilization of the whole GroES structure. The NMR titration in 3 mM ATP also indicated that the *K*_b_ value for the binding between GroES and SR1 increased more than tenfold as compared with the *K*_b_ value in 3 mM ADP. These results will be discussed in relation to the structure and mechanisms of the chaperonin GroEL/GroES complex.

## Materials and methods

### Materials

Wild-type *E*. *coli* GroES and its ^15^N-labeled form were expressed in *E*. *coli* cells BL21(DE3) using the expression plasmid pETESwild [16]. The expression and purification were done as described previously [17].

The expression plasmid of a single-ring mutant (SR1) of GroEL, in which four mutations (R452E, E461A, S463A and V464A) were introduced [11], was constructed from a wild-type GroEL expression plasmid, pEL-WT [18, 19]. The expression and purification of SR1 were done as described previously [19]. The purified SR1 was stored at –20°C in Tris buffer (50 mM Tris-HCl, 2 mM EDTA, and pH 7.5), which contained 15% (v/v) glycerol, until use.

The nucleotides ADP and ATP were purchased from Sigma, and ADP was purified by anion-exchange chromatography to remove contaminating ATP [19]. All other reagents were of guaranteed-reagent grade.

The concentrations of GroES and SR1 were determined spectrophotometrically using extinction coefficients, $E_{1\text{cm}}^{1\%}=0.143\;\text{and}\;0.21$, respectively, at 280 nm [20]. The concentrations of ADP and ATP were determined using the same molar extinction coefficient of 15,400 M^−1^cm^–1^ at 260 nm. All pH values reported were pH-meter readings at 25°C.

### NMR measurements

2D ^1^H–^15^N HSQC NMR spectra [21] of ^15^N-labeled GroES titrated with SR1 were recorded on a Bruker AVANCE 500-MHz NMR spectrometer. The ^1^H flip angle was optimized at 75° for the fast HSQC measurement. We acquired 128 transients for each of 32 *t*_1_ points with sweep widths of 1115 Hz in the ^15^N dimension and 4995 Hz in the ^1^H dimension. The ^15^N-labeled-GroES and non-labeled-SR1 solutions were first dialyzed against 20 mM Tris-HCl, 100 mM KCl and 10 mM MgCl_2_ (pH 7.5) to remove glycerol, and the protein concentrations and the concentrations of other components were adjusted so as to obtain the final solution conditions. We prepared six ^15^N-labeled GroES solutions of different concentrations of SR1 rather than titrating the same GroES solution with a highly concentrated SR1 solution, because prolonged incubation of the concentrated SR1 occasionally resulted in aggregation. The final solutions for the NMR measurements contained 3.0 mM ADP (or 3 mM ATP), 20 mM Tris-HCl, 100 mM KCl, 10 mM MgCl_2_, 21.4 μM ^15^N-labeled GroES and the indicated concentration of SR1 (0–42 μM), at pH 7.5. The solutions also contained 10% D_2_O for detecting the lock signal of the NMR instrument.

### Data analysis

All NMR spectra were processed and analyzed by NMRPipe [22] and NMRView [23]. The nonlinear least-squares analysis of the NMR titration data was performed using IGOR Pro Version 6.3 (WaveMetrics, Inc., Lake Oswego, OR).

## Results

### NMR spectra of ^15^N-labeled GroES

**Fig 1:**
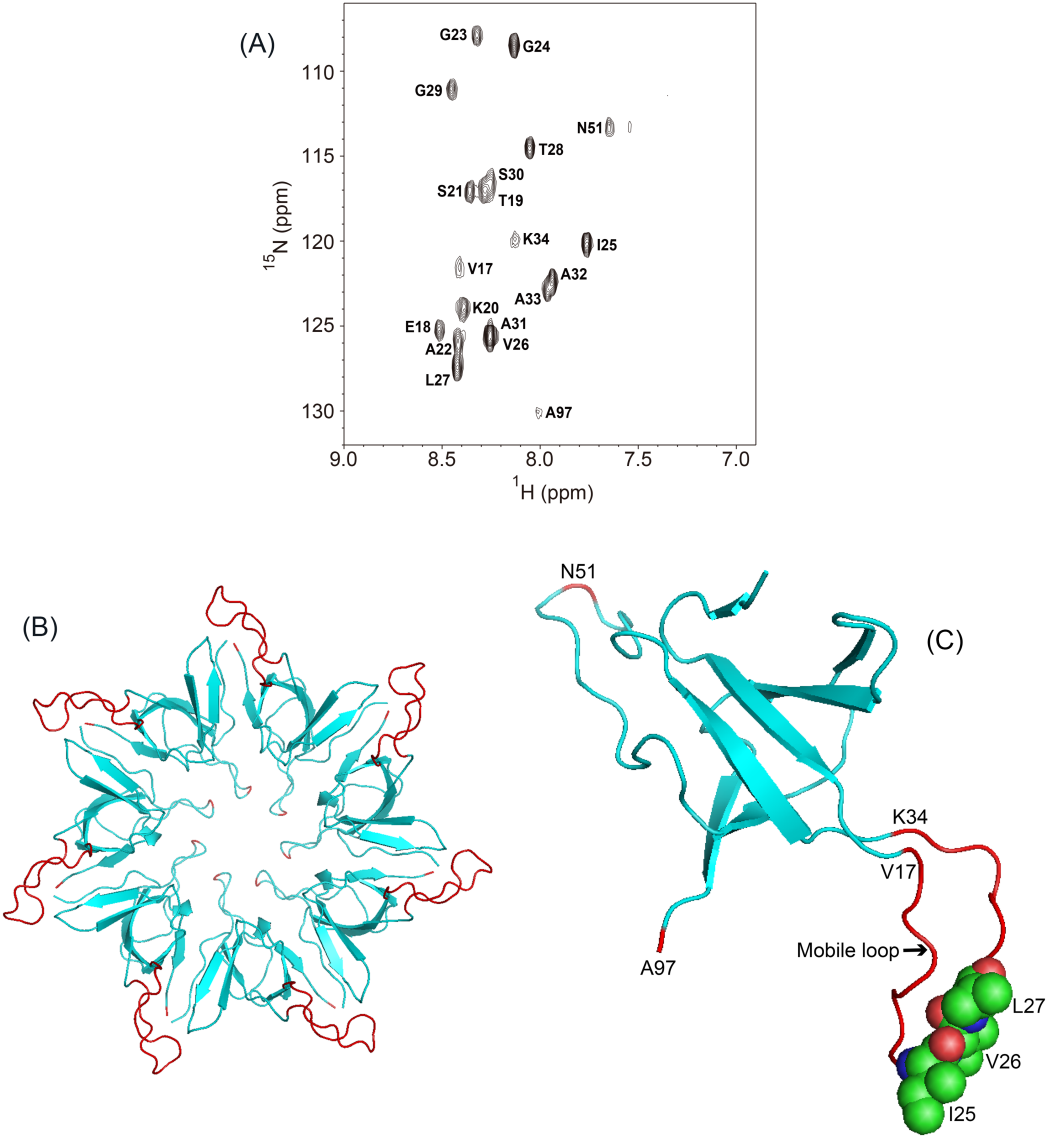
An NMR spectrum and the native three-dimensional structure of GroES. (A) An HSQC spectrum of ^15^N-labeled GroES in 90% H_2_O/10% D_2_O at pH 6.5 and 25°C; and the backbone structures of heptameric GroES (B) and the GroES monomer (C); the flexible mobile loop (residues 17–34), Ala97 and Asn51 are shown in red. In (C), three residues (Ile25, Val26 and Leu27) are shown in a space-filling model. The figures in (B) and (C) were prepared using the GroES portion of the GroEL/GroES/ADP complex (PDB code: 1AON), and drawn by PyMOL (DeLano Scientific).

Fig 1(A) shows a 500-MHz ^1^H–^15^N HSQC spectrum of uniformly ^15^N-labeled GroES in 90% H_2_O/10% D_2_O at pH 6.5 and 25°C. The assignments of the amide-proton signals of the spectrum were taken from Fiaux *et al*. (BioMagResBank entry 7091) [24]. Although native GroES heptamer has a large molecular weight (*M*_*w*_ = 73,000), we observed 20 well resolved ^1^H–^15^N cross-peaks, indicating that these 20 residues were very flexible in the structure, and had a sufficiently short correlation time [25]. According to the above assignments [24], 18 of these residues belong to a flexible mobile loop (residues 17–34) of native GroES [25, 26], and the other 2 residues are Asn51, located at the top of a roof hairpin loop (residues 44–58) [26], and Ala97 at the C-terminus. Fig 1(B) and 1(C) shows cartoon models of the GroES heptamer and its monomer portion, respectively, of the GroEL/GroES/ADP complex (PDB code: 1PF9) [6, 27], and the above 20 residues are shown in red.

### NMR titration of ^15^N-labeled GroES with SR1

**Fig 2:**
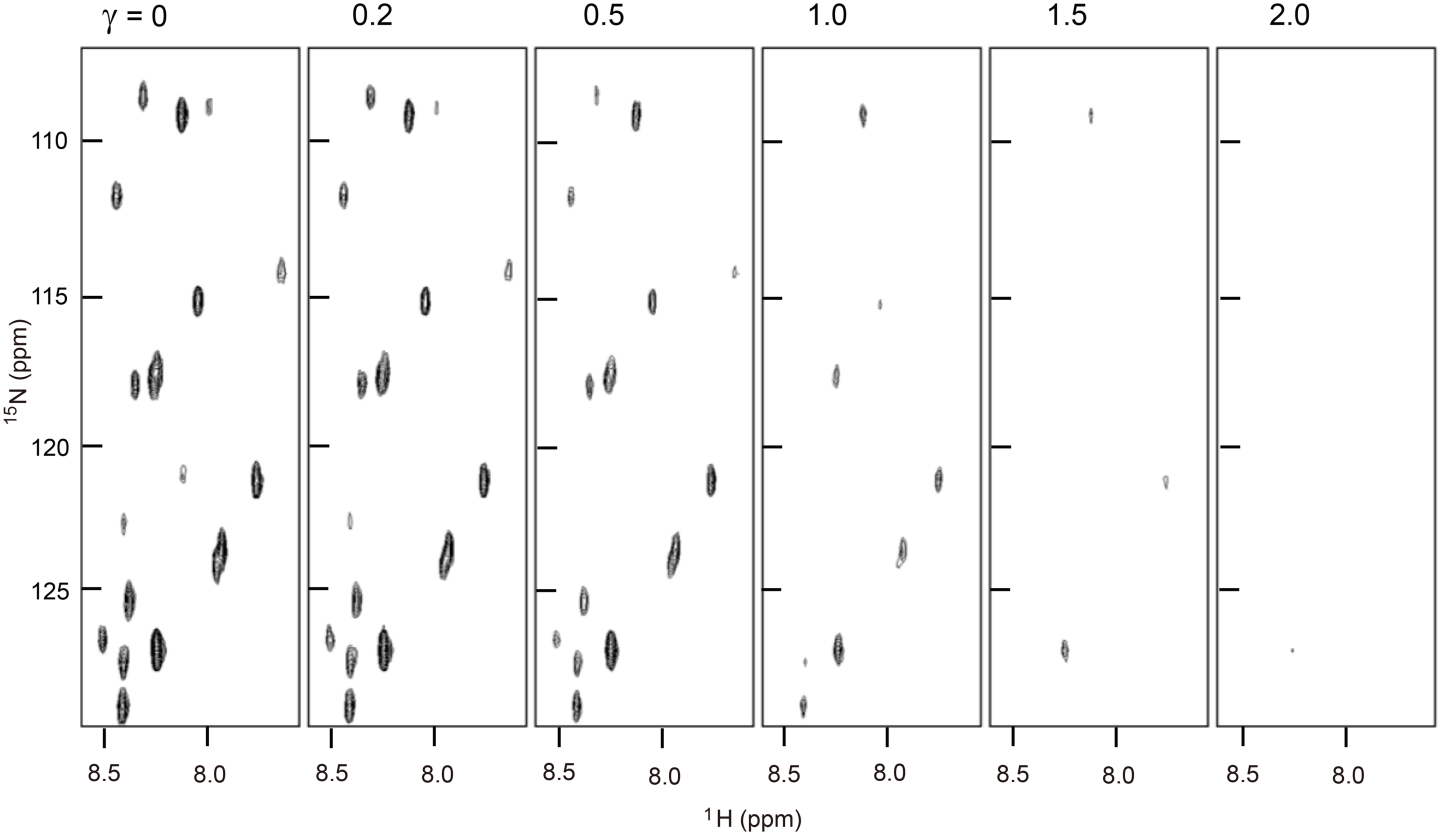
HSQC spectra of ^15^N-labeled GroES as a function of *γ* (= [L]_t_/[P]_t_). The spectra were measured in the presence of 3.0 mM ADP at pH 7.5 and 25°C. [P]_t_ (= 21.4 μM) was kept constant.

We carried out NMR titrations of ^15^N-labeled GroES with SR1 in the presence of 3.0 mM ADP at three different temperatures, 20, 25 and 30°C, at pH 7.5, and the ^1^H–^15^N HSQC spectra of GroES at different molar ratios (*γ*) of SR1 to GroES, *γ* (= [L]_t_/[P]_t_) = 0, 0.2, 0.5, 1.0, 1.5 and 2.0, at 25°C are shown in Fig 2, where [L]_t_ and [P]_t_ represent the total molar concentration of the ligand (heptameric SR1) added to the GroES solution and the total molar concentration of the protein (heptameric ^15^N-labeled GroES) after addition of SR1, respectively, and [P]_t_ was set constant (21.4 μM) throughout. The signal intensities of the 18 residues in the mobile loop as well as Asn51 at the top of the roof hairpin decreased with increasing SR1 concentration. Upon binding by SR1 to GroES, these residues were immobilized, and their NMR cross peaks were broadened, having a large correlation time that was equivalent to the correlation time (~120 ns) of the whole GroES/SR1 complex, and hence they apparently disappeared.

**Fig 3:**
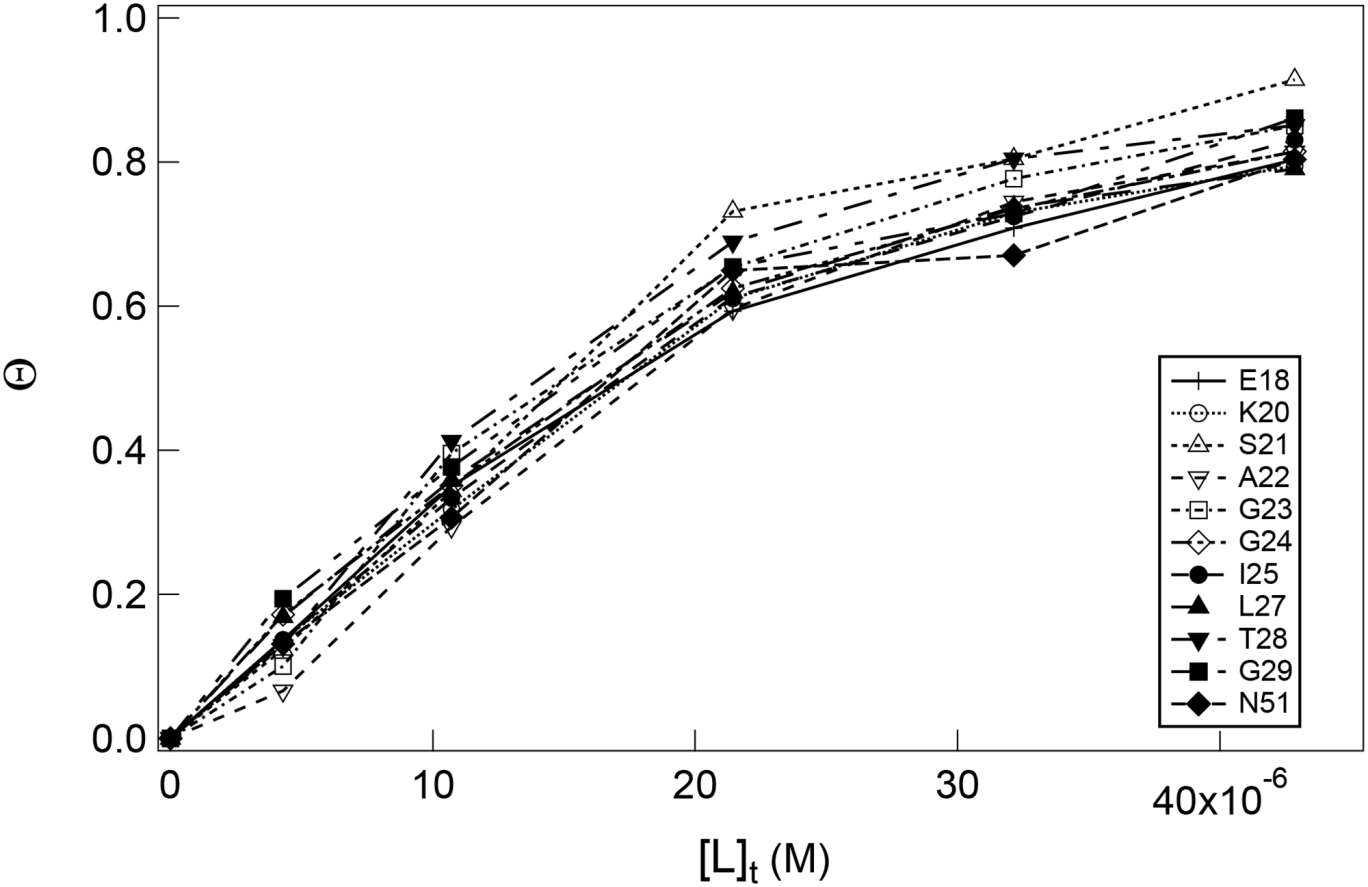
Apparent NMR titration curves of the 11 residues (Glu18, Lys20, Ser21, Ala22, Gly23, Gly24, Ile25, Leu27, Thr28, Gly29 and Asn51) of ^15^N-labeled GroES titrated with SR1. The titration was carried out in the presence of 3.0 mM ADP at pH 7.5 and 25°C. [P]_t_ (= 21.4 μM) was kept constant.

Fig 3 shows NMR titration curves of the 11 residues, Glu18, Lys20, Ser21, Ala22, Gly23, Gly24, Ile25, Leu27, Thr28, Gly29 and Asn51, whose NMR signals were sufficiently strong and well separated (Fig 1(A)); 10 residues are involved in the mobile loop (residues 17–34), while Asn51 is located at the top of the roof hairpin (Fig 1(B) and 1(C)). To compare the signals of the different amino-acid residues, we plotted the apparent fractional saturation, Θ, of the GroES/SR1 complex formation. Θ is represented by the volume ratio as Θ = (*V*_t_ − *V*)/*V*_t_, where *V* is the volume of a cross peak and *V*_t_ is the initial volume of the cross peak in the absence of SR1. As seen from Fig 3, the titration curves of the 11 residues are coincident with each other, strongly suggesting that the SR1 binding to ^15^N-labeled GroES occurs in a single-step cooperative manner. The coincidence of the titration curve of Asn51 with those of the other residues indicates that the immobilization of the structure takes place not only in the mobile loop, which forms the direct binding region recognized by SR1 [25, 27], but also in the whole molecule of GroES.

The single-step binding of the ligand L (SR1) to the protein P (GroES) is represented by the following equation:

$$\text{P}+\text{L}\overset{K_{\text{b}}}{⇄}\;\text{PL}$$ (1)

where PL represents the GroES/SR1 complex, and we assumed a 1:1 binding between GroES and SR1 with a binding constant *K*_b_. It is thus followed by:

$$K_{\text{b}}=\frac{[\text{PL]}}{[\text{P][L]}}$$ (2)

where [P] and [L] are the molar concentrations of GroES and SR1 in the free heptamer state, and [PL] is the molar concentration of the GroES/SR1 complex. However, we knew only the total molar concentrations, [P]_t_ and [L]_t_, of GroES and SR1, respectively, instead of [P] and [L]. The relationships between [P] and [P]_t_ and between [L] and [L]_t_ are given by:

$$[\text{P]}={[\text{P]}}_{\text{t}}-[\text{PL]}$$ (3)

$$[\text{L]}={[\text{L]}}_{\text{t}}-[\text{PL]}$$ (4)

The fractional saturation of the complex formation Θ is expressed as:

$$\Theta =\frac{V_{\text{t}}-V}{V_{\text{t}}}=\frac{{[\text{P]}}_{\text{t}}-[\text{P]}}{{[\text{P]}}_{\text{t}}}=\frac{[\text{PL]}}{{[\text{P]}}_{\text{t}}}$$ (5)

From Eqs (2)–(5), Θ is given as a function of [L]_t_ as:

$$\Theta =\frac{1+K_{\text{b}}{[\text{P]}}_{\text{t}}+K_{\text{b}}{[\text{L]}}_{\text{t}}-{\left\{{\left(1+K_{\text{b}}{[\text{P]}}_{\text{t}}+K_{\text{b}}{[\text{L]}}_{\text{t}}\right)}^{2}-4K_{\text{b}}^{2}{[\text{P]}}_{\text{t}}{[\text{L]}}_{\text{t}}\right\}}^{\frac{1}{2}}}{2K_{\text{b}}{\left[P\right]}_{\text{t}}}$$ (6)

where [P]_t_ is a constant ([P]_t_ = 21.4 μM), and [L]_t_ is an independent variable in the present case. For a more general case, in which there are *n* independent SR1-binding sites in GroES, Eq 6 is rewritten as:

$$\Theta =\frac{1+nK_{\text{b}}{[\text{P]}}_{\text{t}}+K_{\text{b}}{[\text{L]}}_{\text{t}}-{\left\{{\left(1+nK_{\text{b}}{[\text{P]}}_{\text{t}}+K_{\text{b}}{[\text{L]}}_{\text{t}}\right)}^{2}-4nK_{\text{b}}^{2}{[\text{P]}}_{\text{t}}{[\text{L]}}_{\text{t}}\right\}}^{\frac{1}{2}}}{2nK_{\text{b}}{\left[\text{P}\right]}_{\text{t}}}$$ (7)

**Fig 4:**
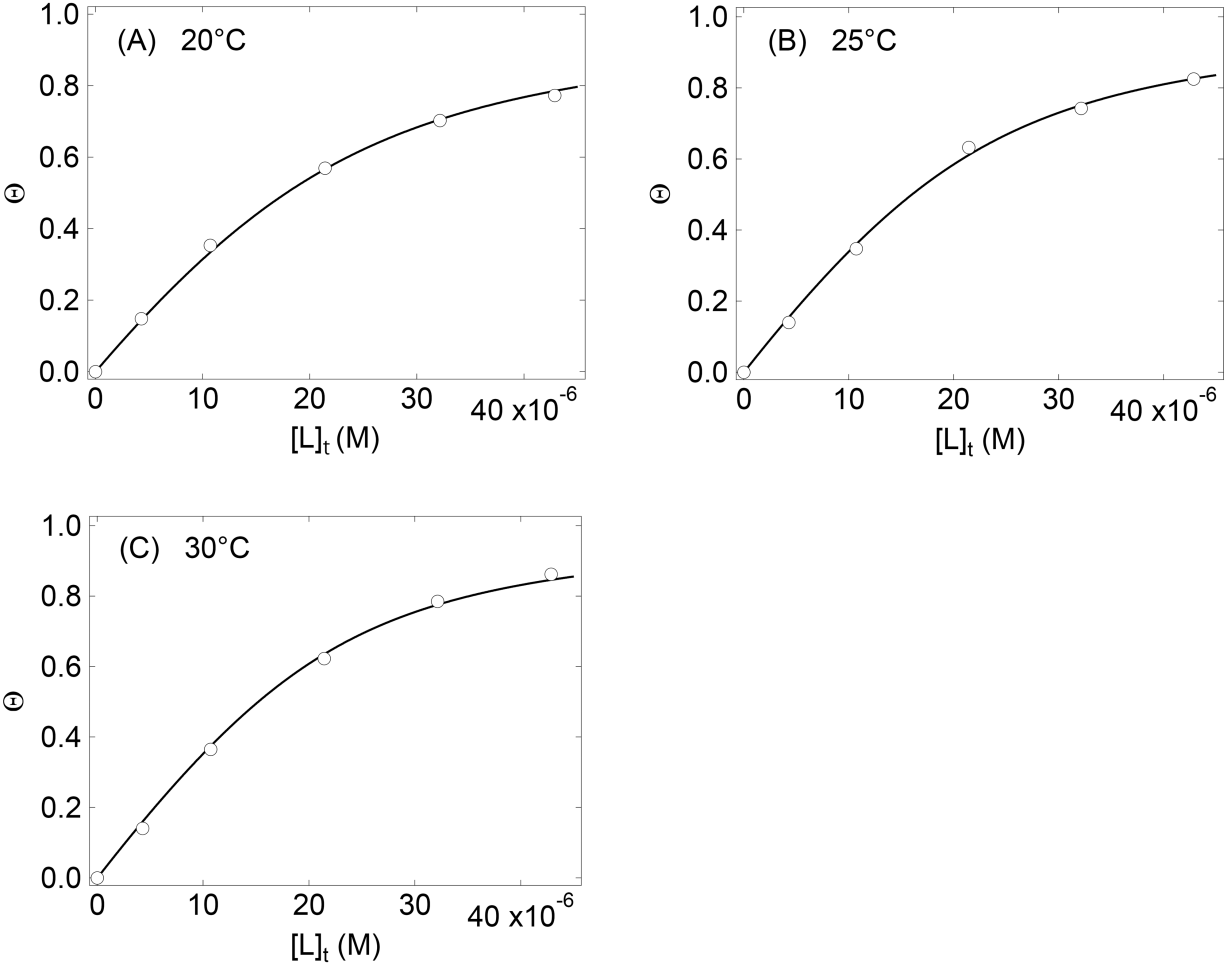
Binding isotherms of ^15^N-labeled GroES titrated with SR1 at three different temperatures. The experiments were carried out in the presence of 3.0 mM ADP at pH 7.5, at 20°C (A), 25°C (B) and 30°C (C). [P]_t_ (= 21.4 μM) was kept constant. The solid lines are the theoretical curves best fit to the experimental data using Eq 6.

Fig 4 shows the binding isotherms of ^15^N-labeled GroES titrated with SR1 in the presence of 3 mM ADP at three different temperatures. The Θ values shown are those obtained by averaging the values for the 11 residues shown in Fig 3. The solid lines in Fig 4 are the theoretical curves best-fitted to Eq 6 by using the nonlinear least-squares analysis, and the best-fit *K*_b_ values thus obtained are summarized in Table 1. The theoretical curves show excellent agreement with the experimental data in Fig 4, indicating that the single-step binding model of Eq 1 well represents the SR1 binding to GroES under the present conditions.

**Table 1:** The *K*_b_ values between GroES and SR1.

Temp. (°C)	*n*	*K*_b_ (M^−1^)	Δ*H* (kJ/mol)	Δ*S* (J/mol/K)	SR1 conc. (μM)	GroES conc. (μM)	Conditions
		×10^5^					
20	1*	1.41±0.05	35.1±4.8	219±10	0–42	21.4	3.0 mM ADP, 0.1 M KCl, 10 mM MgCl_2_, pH 7.5
25		1.89±0.10			0–42	21.4	
30		2.23±0.13			0–42	21.4	
		×10^6^					
25	1*	2.35±0.99			0–42	21.4	3 mM ATP, 0.1 M KCl, 10 mM MgCl_2_, pH 7.5

*The stoichiometric number *n* is fixed (*n* = 1).

**Fig 5:**
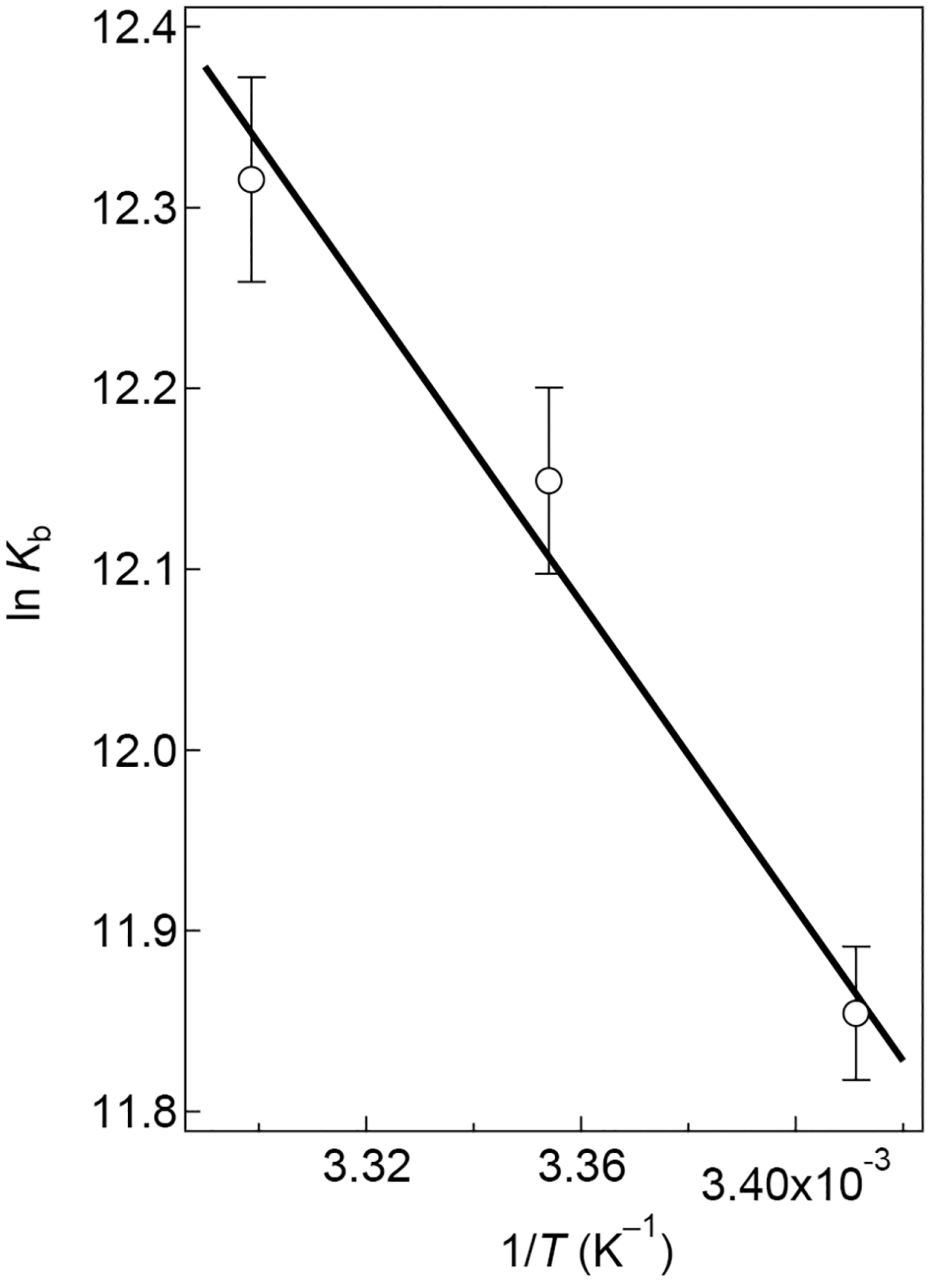
The van't Hoff plot for the GroES–SR1 binding reaction in the presence of 3.0 mM ADP. The solid line indicates the theoretically best fit to the following equation: ln *K*_b_ = *a* + *b*(1/*T*), where *a* and *b* are constant, fitting parameters. The best-fit parameter values obtained by the least-squares analysis were *a* = 26.3±1.2 and *b* = (−4.23±0.58)×10^3^ K. The Δ*H* and Δ*S* for the GroES–SR1 binding are given by: Δ*H* = −*bR*, and Δ*S* = *aR*, where *R* is the gas constant.

From Table 1, *K*_b_ increases with an increase in temperature, indicating that there is a significant enthalpy increase upon the GroES/SR1 complex formation. On the assumption of the single-step binding of SR1 to GroES (Eq 1), the reaction enthaply Δ*H* can be estimated by the van't Hoff equation:

$$\Delta H=-R\frac{d\;\text{ln}\;K_{\text{b}}}{d\left(1/T\right)}$$ (8)

where *R* is the gas constant, and *T* the absolute temperature. Fig 5 shows the plot of (ln *K*_b_) vs. 1/*T* (the van't Hoff plot), from which we have estimated the Δ*H* and the reaction entropy Δ*S* at 35±5 kJ/mol and 219±10 J/mol/K, respectively. The positive Δ*H* and Δ*S* upon the GroES/SR1 complex formation indicate that the binding reaction is an entropically driven process.

**Fig 6:**
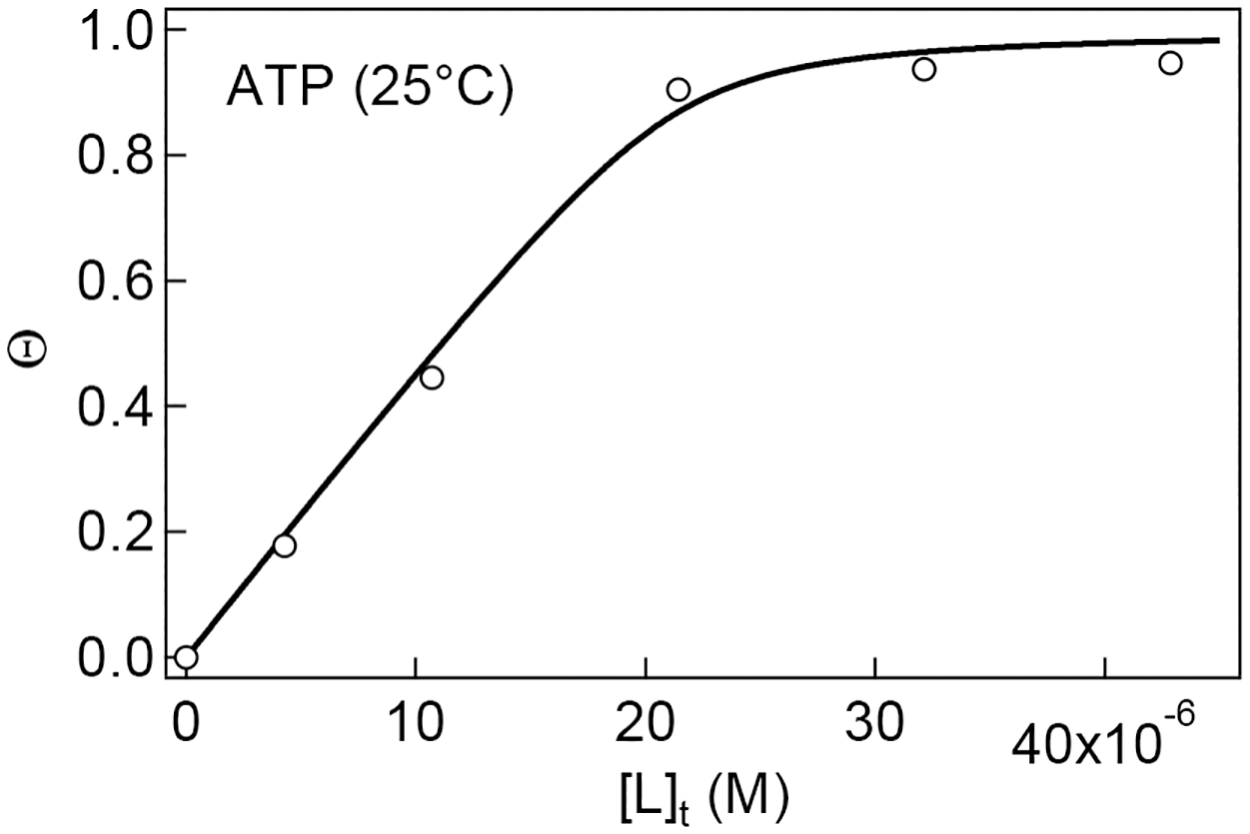
A binding isotherm measured by the cross-peak intensities of ^15^N-labeled GroES titrated with SR1 in the presence of 3 mM ATP. The titration was carried out at pH 7.5 and 25°C. [P]_t_ (= 21.4 μM) was kept constant. The solid line is the theoretical curve best fit to the experimental data using Eq 6.

To compare the binding affinity of the ADP-bound SR1 to GroES, shown above, with the affinity of the ATP-bound SR1 to GroES, we carried out the NMR titration experiments in the presence of 3 mM ATP at pH 7.5 and 25°C. Because of the ATP hydrolase activity of SR1, such an experiment might be affected by the ATP hydrolysis. However, the ATP hydrolase activity of SR1 is strongly inhibited by binding to GroES [11], and hence the titration of GroES with SR1 in 3 mM ATP will give us meaningful results of the SR1–GroES binding in the ATP state. The resultant binding isotherm is shown in Fig 6. The binding isotherm is steeper than that in Fig 4, and there is a kink at the equimolar concentration (21 μM) of SR1, indicating that SR1 binds much more strongly to GroES in the presence of ATP. The nonlinear least-squares fitting of the data in Fig 6 to Eq 6 gave a *K*_b_ value of 2.4×10^6^ M^−1^, more than 10 times stronger than the corresponding value for the ADP-bound SR1 (Table 1).

## Discussion

We studied the binding between GroES and SR1 by the NMR titration of ^15^N-labeled GroES with SR1 in the presence of 3 mM ADP at pH 7.5. We assumed that the binding was well-represented by a single-step cooperative binding with a binding stoichiometry of one (Eq 1). The binding reaction was endothermic with a positive Δ*H* (= 35 kJ/mol) and a positive Δ*S* (= 219 J/mol/K), indicating that the reaction was solely driven by the entropy increase. As far as we are aware, this is the first report on the determination of these thermodynamic parameters of the chaperonin complex formation. The NMR titration, carried out in the presence of 3 mM ATP, also indicated that the binding constant between GroES and SR1 increased more than tenfold as compared with the binding constant in 3 mM ADP (Table 1). These results should thus be discussed in relation to the structure and molecular mechanisms of the chaperonin GroEL/GroES complex. In the following, we will discuss (1) immobilization of the GroES molecule upon binding to SR1 as revealed by the NMR titration experiments, (2) the entropic nature and the molecular mechanisms of GroES–SR1 binding, and finally (3) ATP-induced enhancement of the binding between GroES and SR1.

### Immobilization of GroES upon binding to SR1

Although native GroES is a large protein assembly with a molecular weight of 73,000, we observed well-resolved cross peaks of 20 residues in the ^1^H–^15^N HSQC spectrum of ^15^N-labeled GroES. These 20 residues, consisting of residues 17–34 in the mobile-loop segment [25, 26], Asn51 located at the top of the roof-hairpin loop [26], and Ala97 at the C-terminus of GroES, were thus very flexible in the native structure. Landry *et al*. [25] first observed the cross peaks of residues 17–32 of GroES in homonuclear 2D NMR spectra (TOCSY and NOESY), and named this polypeptide segment the mobile loop, because the sharp NMR cross peaks indicated substantial mobility of the peptide segment. The X-ray crystallographic analysis of stand-alone GroES also revealed that the electron density of the mobile-loop segment (residues 16–32) was not observed in six of the seven GroES subunits [26], indicating the high flexibility of this segment in native GroES. The present study has further indicated that in addition to the mobile-loop segment, two residues, Asn51 and Ala97, exhibit clear cross peaks in the HSQC spectrum (Fig 1(A)), demonstrating that the flexible nature of the structure is not localized in the mobile-loop segment, but is also present in the other portions, the top of the roof hairpin and the C terminus of the GroES molecule. The roof formed by the seven roof-hairpin loops of GroES subunits is not fully closed, but there is a hole of about 5 Å in diameter at the center of the domed roof, and Asn51 is just located at the outer orifice of this central hole [26]. Our recent study on the hydrogen/deuterium-exchange behavior analyzed by 2D NMR and dimethylsulfoxide-quenched exchange methods has also demonstrated the flexible nature of GroES [17]. Although the hydrogen-exchange protection factors of the most highly protected amide protons of GroES were on the order of 10^6^–10^7^, comparable in magnitude to those observed in typical globular proteins, the number of these most highly protected amide protons was only 10 per monomer unit of GroES, significantly smaller than the numbers reported for other small globular proteins of similar size. The most highly protected amide protons are located in the central hydrophobic core formed by an irregular β-barrel and in the subunit–subunit interface, and hence the other portions of the GroES heptamer are flexible in terms of the hydrogen-exchange behavior [17]. Nishida *et al*. [28] have also shown by ^13^C NMR of GroES with tyrosine- and histidine-specific ^13^C-labeling that GroES contains divergent local mobility throughout its structure, which is consistent with the flexible nature of the GroES oligomer.

We titrated ^15^N-labeled GroES with SR1 (Fig 2). Upon binding of SR1 to GroES, the cross peaks apparently disappeared, and hence the residues that had shown the cross peaks were immobilized. The NMR titration curves of the 11 residues, Glu18, Lys20, Ser21, Ala22, Gly23, Gly24, Ile25, Leu27, Thr28, Gly29 and Asn51, whose cross peaks were sufficiently strong and well-separated, exhibited coincident titration curves (Fig 3), indicating that the SR1 binding to GroES occurred in a cooperative single-step manner. Because the mobile-loop segment of GroES is the binding site recognized by GroEL [27], the simultaneous immobilization of the residues in the mobile loop is reasonably expected. However, Asn51, which is located at the side of GroES opposite the mobile loop (Fig 1(B) and 1(C)), was also simultaneously immobilized, leading to the conclusion that the immobilization took place in a cooperative manner in almost the whole molecule of bound GroES. A previous hydrogen/deuterium-exchange study of the SR1/GroES complex has also shown that the residues in the mobile loop, which are not highly protected in free heptameric GroES, are strongly protected in the complex with a protection factor of 10^5^–10^6^ [29]. The TROSY (transverse relaxation-optimized spectroscopy) spectra of free and bound GroES, reported by Fiaux *et al*. [24], also showed that after adding one equivalent of either SR1 or GroEL, nearly all of the TROSY resonances observed with free GroES disappeared, because of the simultaneous immobilization of the bound GroES. However, the cross-peak for Ala97 was still visible in the GroEL/GroES complex, suggesting that it remains flexibly disordered while bound to the chaperonin [24]. These previous studies are thus fully consistent with the above conclusion in the present study.

### Entropic nature and molecular mechanisms of GroES–SR1 binding

The present results indicate that the SR1–GroES binding is endothermic with significantly positive Δ*H* and Δ*S* (Table 1). Such entropically driven behavior is a characteristic of the hydrophobic interactions at room temperature, and the Δ*S* is positive because the interactions release the water molecules that have been restrained by the hydrophobic surfaces until their coalescence [30–32]. From the X-ray crystal structure of the GroEL/GroES/(ADP)_7_ chaperonin complex reported by Xu *et al*. [27], the GroEL–GroES interface consists of mostly hydrophobic aliphatic side chains, including Ile25, Val26 and Leu27 in the mobile loop of GroES (see Fig 1(C)), and Leu234, Leu237 on helix H (residues 234–243) and Val264 on helix I (257–268) of GroEL, and this fully supports the above conclusion that the SR1–GroES binding is promoted by hydrophobic interactions.

Interestingly, the interactions between model substrate proteins and free tetradecameric GroEL were also endothermic and driven entropically, and hence promoted by hydrophobic interactions [33–35]. This behavior is thus similar to that observed in the SR1–GroES binding in the present study. The binding site for substrate proteins in GroEL was investigated by a protein engineering and mutational analysis [36], X-ray crystallography [37, 38], fluorescence analysis [39], NMR spectroscopy [40, 41] and cryo-electron microscopy [42]. The binding site was essentially identical to the binding site for the mobile loop of GroES, and hence located at a groove between helix H and helix I of GroEL, although the substrate proteins were also bound to an extended segment of residues 199–203 under helix I [42]. The identity of the binding sites for GroES and the substrate proteins is thus fully consistent with the similarity of the thermodynamic behavior between the GroES–SR1 binding and the substrate–GroEL binding mentioned above.

### ATP-induced enhancement of the binding between GroES and SR1

The NMR titration experiments were also carried out in 3 mM ATP. The results have shown that the binding affinity between GroES and SR1 is about ten-fold stronger in ATP than in ADP (Table 1). Previous studies have also shown that the GroEL/GroES/ATP complex, which is resistant to exposure to 0.4 M guanidinium chloride, is more stable than the GroEL/GroES/ADP complex [6, 43]. The present results further provide a quantitative measure of the stabilization by ATP, and the ratio of the *K*_b_ values shown in Table 1, i.e., (2.35×10^6^)/(1.89×10^5^) = 12.4, gives an additional stabilization free energy of −6.2 kJ/mol (= −*RT* ln 12.4) at 3 mM ATP as compared with the standard free energy change, −30.1 kJ/mol (= −*RT* ln 1.89×10^5^), of the GroES–SR1 binding in 3 mM ADP at 25°C. The enhancement of the affinity for GroES in ATP may be important for efficient operation of the chaperonin cycle, in which the GroES binds selectively to the ATP-bound new *cis* ring of GroEL [6, 43].

## Conclusions

1. Native heptameric GroES is a highly flexible protein, and the amide proton signals of not only the flexible mobile loop (residues 17–34) but also Asn51 at the top of the roof hairpin loop and Ala97 at the C terminus were well resolved in the ^1^H–^15^N HSQC spectrum (Fig 1(A)).
2. The binding of SR1 to GroES immobilized most of these flexible residues in a single-step, cooperative manner as evidenced by the simultaneous disappearance of the HSQC cross peaks of the 11 residues, including the residues in the mobile loop and Asn51, in 3 mM ADP at pH 7.5, although the cross peak of Ala97 was still visible in the SR1-bound state.
3. There are similarities in the thermodynamic parameters (positive Δ*H* and positive Δ*S*) between the GroES–SR1 binding and the substrate–GroEL binding previously studied. This may reflect the fact that the GroES mobile loop and the substrate polypeptides are recognized by the same binding site of GroEL (or SR1).
4. The NMR titration carried out in 3 mM ATP was compared with the titration in 3 mM ADP. The binding affinity between GroES and SR1 was 12.4-fold stronger in ATP than in ADP. The enhancement of the affinity of SR1 for GroES in ATP may be important for efficient operation of the chaperonin cycle, in which the GroES binds selectively to the ATP-bound new *cis* ring of GroEL.
